# The animal sensorimotor organization: a challenge for the environmental complexity thesis

**DOI:** 10.1007/s10539-017-9565-3

**Published:** 2017-02-16

**Authors:** Fred Keijzer, Argyris Arnellos

**Affiliations:** 1 Department of Theoretical Philosophy, University of Groningen, Groningen, The Netherlands; 2 Department of Logic and Philosophy of Science, IAS-Research Centre for Life, Mind and Society, University of the Basque Country (UPV/EHU), Donostia-San Sebastián, Spain

**Keywords:** Environmental complexity thesis, Early nervous systems, Skin brain thesis, Sensorimotor system, Epithelia, Multicellular organization

## Abstract

Godfrey-Smith’s environmental complexity thesis (ECT) is most often applied to multicellular animals and the complexity of their macroscopic environments to explain how cognition evolved. We think that the ECT may be less suited to explain the origins of the animal bodily organization, including this organization’s potentiality for dealing with complex macroscopic environments. We argue that acquiring the fundamental sensorimotor features of the animal body may be better explained as a consequence of dealing with internal bodily—rather than environmental complexity. To press and elucidate this option, we develop the notion of an animal sensorimotor organization (ASMO) that derives from an internal coordination account for the evolution of early nervous systems. The ASMO notion is a reply to the question how a collection of single cells can become integrated such that the resulting multicellular organization becomes sensitive to and can manipulate macroscopic features of both the animal body and its environment. In this account, epithelial contractile tissues play the central role in the organization behind complex animal bodies. In this paper, we relate the ASMO concept to recent work on epithelia, which provides empirical evidence that supports central assumptions behind the ASMO notion. Second, we discuss to what extent the notion applies to basic animal architectures, exemplified by sponges and jellyfish. We conclude that the features exhibited by the ASMO are plausibly explained by internal constraints acting on and within this multicellular organization, providing a challenge for the role the ECT plays in this context.

## Introduction

In 1996 Peter Godfrey-Smith published his book *Complexity and the Function of Mind in Nature*. Here, he first put forward his influential *environmental complexity thesis* (ECT) as a general principle about the evolution of cognition: “The function of cognition is to enable the agent to deal with environmental complexity.” This succinct phrase reflects an insightful and wonderfully intricate set of ideas and reflections on the ways organisms derive advantages by using cognitive capacities to coordinate their behavior with the state of the environment (Godfrey-Smith 1996a, b, 2002). Here we will focus on, and challenge, the idea that the ECT provides a sufficient account for the evolutionary origins of animal cognition. We treat the ECT as an externalist proposal that prioritizes environmental heterogeneity as the key feature for the evolution of cognition. This does not imply that we interpret Godfrey-Smith’s position as a form of simple asymmetric externalism (properties of the environment fully explain the properties of the organic system, but not the other way around) a position that he criticizes himself. Godfrey-Smith’s position combines internalist and externalist elements, and the same goes for our own view defended below. The difference with Godfrey-Smith concerns the emphasis and relative importance that we respectively place on these organismal and environmental aspects. Treating his position as an externalist one merely reflects that for the ECT the environment plays a more prominent role than the agent.

Our focus will be on the notions of agent and environment that both feature in the ECT. These notions come together as an agent implies some environmental context in or on which it operates, an issue that we will address below. While Godfrey-Smith (2002, in press) discusses the possibility of ‘proto-cognition’ in bacteria, plants and less complex animals, the default target of the ECT are complex animals and the environment they inhibit. Complex animals—in particular humans—are after all the standard targets when we think of cognition and the mind. In these cases, the ECT applies to animal—and thus multicellular—agents acting on and reacting to a macroscopic environment composed of recognizable objects (trees, grass, predators etc.) and various media available for locomotion (e.g. water, soil and various surfaces). We claim that neither animals—being multicellular organizations—nor the macroscopic environment in which they act can be taken as a self-evident starting-point for the evolution of cognition.

O’Malley (2014) recently sketched the far-reaching philosophical implications of current microbiology and argued that life should be considered *microbial by default*, while multicellular *macrobial* forms are specializations that only occur in a limited number of lineages. The implication is that animals and other multicellulars are special cases the occurrence of which requires additional explanations on top of a more basic cellular organization. Even if you find this claim too strong, the fact remains that animals consist of complex and peculiar multicellular organizations that in their modern form only became visible with the Cambrian Explosion, about 542 Ma, in contrast to unicellular life that has been ongoing since about 3.5 Ga. Trestman (2013) used the phrase *complex active bodies* to refer to these new entities that he describes with a cluster of related properties: “(1) articulated and differentiated appendages; (2) many degrees of freedom of controlled motion; (3) distal senses (e.g., “true” eyes); (4) anatomical capability for active, distal-sense-guided mobility (fins, legs, jet propulsion, etc.); and (5) anatomical capability for active object manipulation (e.g., chelipeds, hands, tentacles, mouth-parts with fine-motor control)” (p. 81). Such properties are exactly those that allow animals to act on the macroscopic environment of which we—as humans—have firsthand experience. It is an important evolutionary question how and why these modern animal features arose.

In contrast to work on the Cambrian Explosion of complex animal bodies (Marshall 2006), much less is written, or understood, about the prequel of this key event (Keijzer 2015). Trestman rightly stresses that ‘complex active bodies’ require additional, cognitive features to make them operative. He proposes the initial presence of “a cognitive toolkit for pickup, tracking, and use (for control of behavior) of certain information from the environment” (2013, p. 89), which would be realized by some form of nervous system. However, where did such a convenient neural tissue organization and its characteristics come from in the first place? Trestman acknowledges this problem when he writes that these requirements “do not simply follow from selection pressure due to predation or competition, but requires some (unknown) set of preadaptations in neural control architecture, which must be to some extent fortuitous—there is no foresight in evolution” (p. 91). If this claim about preadaptations is right, and we think it is, this spells a difficulty for the ECT at its very starting point: Multicellular agents but also the environments in which they act are themselves in need of an explanation (e.g. Arnellos and Moreno 2015).

The evolutionary transition from a collection of cells to active animals has so far remained outside of the scope of the ECT. However, this transition is not clear cut at all. Bonner (2000) mentions a minimum of 13 independent transitions to multicellularity, others discuss an even higher count of 25 (O’Malley et al. 2013), the animal case being only one of these. None of the other cases did turn into anything even remotely resembling the free-moving life style that is typical for complex animals. Thus, the evolution of an animal multicellular organization, in particular macroscopic, freely moving agents, cannot be taken as a self-evident, evolutionary next step that is to be expected when multicellular lifeforms arise.

In this paper, we address the question how and why this evolutionary transition might have taken place and the role of early nervous systems therein. Our discussion comes close to some of Godfrey-Smith’s own recent work (Godfrey-Smith, in press; Jékely et al. 2015a), which recognizes a distinction between Internal Coordination (IC) and Input–Output (IO) views on the role of (early) nervous systems. An IC view highlights the need to “induce and coordinate activity internal to large multicellular organizations” (Jékely et al. 2015a, p. 1) and can be contrasted to a more environmentally oriented IO view (ibid.) that is most easily aligned with the ECT. While both views can, and must, be combined, their relative importance is an issue of discussion. Here, we will press the claim that IC issues provide the key to explaining the transition to the modern animal organization.

The main problem we explore concerns the features that are required to build and constitute a multicellular organization that is sensitive to its own and its environment’s macroscopic organization. There is an old metaphor for the mind where the world acts as a stamp that imposes its structure on our memory, envisioned as a wax tablet. This metaphor assumes a structure, a wax tablet, which is sensitive to the stamp and retains the information it imposes once the stamp is removed. While sensitivity to environmental features at a biomolecular and unicellular scale is essential to maintain any living organization, in animals with active bodies an organization is present that has such *sensitivity at the multicellular level*. True (image forming) eyes are a good example. Here the spatial visual structure is somehow taken up by the animal, in a way that is not organized within any individual cell, but spread out across several cells where the position of these cells with respect to one another becomes central. In this case, the individual cells are no longer the single level at which the organism interacts with ‘the’ environment but the multicellular organization brings in its own additional organization allowing the environment to expand into the macroscopic configurations that we ourselves inhabit.

Where does this multicellular organization come from and how does it function? Recently, this problem has been targeted by an IC proposal concerning early nervous systems (Keijzer et al. 2013). This ‘skin brain thesis’ proposes that the coordination of *contractile epithelia* for motility formed the key adaptive feature behind the earliest nervous systems that evolved, presumably long before the Cambrian Explosion (Erwin 2015; Wray 2015; but see also Budd 2015). In addition, Keijzer (2015) argued that such a spatially extended proto-neural and contractile system also provides the basic ingredients for a multicellular *sensing* organization: Self-induced epithelial contraction combined with stress-induced cellular feedback can connect macroscopic patterns across a multicellular epithelium to specific events at a cellular level. The result can be envisioned as a small slug, consisting of connected contractile cells, that controls its overall body-shape in a reversible way by manipulating the contraction levels across its body in a way that is sensitive to both self- and externally induced changes in these contraction levels at a cellular level. A configuration like this could solve the question how macroscopic happenings that extend across a multicellular organization became able to ‘talk’ to, and in this way become coordinated with, intracellular events. Metaphorically, a contractile biomolecular organization constituting the now active ‘wax tablet’ has acquired a means that enables it to tell apart the different macroscopic patterns it actively stamps on.

As argued by Keijzer (2015), such a tissue organization provides a foundation for a sensorimotor organization that is specific for animals that use contractile tissue to generate motility. Keijzer referred to this as an *animal sensorimotor organization* or ASMO. An ASMO is claimed to be present in all animals with nervous systems and muscles, and it provides a source for the capacity to pick up and use macroscopic patterns of environmental information that became typical for Cambrian style motile animals. In this paper, we develop the ASMO notion in some detail by discussing its relation to *epithelia*. Epithelia constitute the basic and most typical tissue organization exhibited by animals (Tyler 2003; Leys and Riesgo 2012). Here, we discuss how an epithelial organization and its properties can be cast as the key enabling factor for the evolution of the ASMO and also how a focus on the epithelial organization integrates the ASMO more closely to development and physiology; all essential features of the organizational basis of complex animals (Arnellos and Moreno 2015, 2016).

Some may wonder whether animal bodies can be considered to be part of the cognitive domain. However, within embodied approaches to cognition, the notion of cognition is already applied in a wide sense (e.g. Barrett 2011; Chemero 2009) and the same goes for ongoing discussions on bacterial (Lyon 2015) and plant cognition (Calvo and Keijzer 2011). In this context, the foundations of the animal sensorimotor organization easily fulfils the criteria for a minimal reading of cognition.

Without addressing the ECT directly here, the ASMO notion sets up a challenge for the ECT nevertheless. While the ECT stresses environmental complexity as the main driving force towards complex forms of intelligent behavior by animals, the ASMO notion highlights the prior importance of the (internal) multicellular organization as a precondition for the macroscopic environment inhabited by animals to become accessible for these animals. The challenge for the ECT is whether the origins of the animal sensorimotor organization—on which the ECT depends—can also be explained in terms of *environmental* complexity or whether an IC account has the better cards here.

The paper has the following structure. In the section “The animal sensorimotor organization (ASMO)”, we give a short description of the ASMO notion. “Epithelia and their features” discusses the characteristics of epithelia, while in “Epithelia and the animal sensorimotor organization” we sketch how epithelia provide a very suitable foundation for the characteristics of the ASMO. In “Two model animal organizations: Porifera and Cnidaria”, the features of the ASMO are related to typical characteristics of Porifera (sponges) and Cnidaria (such as jellyfish). Finally, “Bodily complexity meets environmental complexity” provides a short discussion how the discussion on the ASMO sets up a challenge for the ECT.

## The animal sensorimotor organization (ASMO)

The ASMO is a solution to a problem that is not always clearly perceived: Acquiring a biological organization that is sensitive to macroscopic environmental features, including the macroscopic features of the body itself. The problem derives from the transition of single cells capable of a sensorimotor interaction with their environment to a multicellular organization doing the ‘same’ but at a significantly larger scale. As long as we discuss this interaction only in terms of input and output—terms that can be described in many different ways—it is not obvious that there are major organizational changes involved in this switch (Keijzer 2015). One important reason seems to be the word ‘brain’ itself. The concept of a brain is often used like that of a material mind that can handle environmental descriptions in many different forms without needing a clear account how this tissue might achieve such a feat. While it is obvious that brains are actually capable of performing this task, there are two important questions here: (1) How do nervous systems function? (2) How could any particular tissue configuration have acquired this ability? Natural selection does not fulfil this exploratory burden on its own as it needs traits to work on (see for instance Arnellos and Moreno (2012) for a detailed discussion of the organizational complexity of protocells). The issue here is how various traits—like sensing and motion control—that are standardly used with respect to brains originated at a very early and basic level in animal tissues.

Multicellular organizations—most notably animals, plants and fungi—organize the way in which they act on and react to their communal environment in various ways. The focus here is on the organization that has evolved within the animal lineage and given rise to their ‘complex active bodies’ as mentioned above. The central idea behind the notion of an ASMO is that complex animal senses did not evolve by stacking individual sensory cells together to form static sensory arrays. Static arrays are implausible for early animals that were soft-bodied and deformable, in particular for the basic sense of touch. Therefore, instead of interpreting animal sensing as an input process, the ASMO notion claims that animal sensing derives from a slug-like organization of cells that are physically connected to one another and capable of contracting. By being physically connected, cells transmit the generated tensions across the whole slug-body. The generated changes in tension and shape across the slug-body can be used as a differentiated signal for the individual cells and their intra-cellular signaling, which in turn influences the slug-body again. Direct physical contact with the environment would influence the organism by impinging on the dispersal of tensions across the slug-body. This configuration distinguishes the ASMO from an input–output system with its clear conceptual distinction between an internal and an external domain. We propose that the ASMO constitutes an essential preadaptation that became the platform for complex sensors, effectors and nervous systems.

The notion of the ASMO derives from an account of the evolution of the first nervous systems (ibid.). This account centers on an idea of Pantin (1956) who stresses the need to coordinate the *animal behavior machine*: muscle. Motility induced by cilia can be used both by single cells and multicellular organisms, and is relatively easy to control (Jékely 2011; Elgeti and Gompper 2013). However, as argued by Pantin, the switch to contraction-based motility—which proved to be an essential step for the later evolution of complex, large animals—required more complex forms of coordination suggesting an important role for early nervous systems. Roughly, to squirt water from a balloon-like body, the animal will have to contract a significant portion of this body and control its body opening at the same time. This involves a fast way to synchronize and coordinate activity across sheets of physically connected cells. It is interesting to note here that nervous systems and muscle always come together in extant animals. Thus, in this internal coordination (IC) account, contractile and reversible changes in body-shape constitute the key phenomenon for early nervous systems. Most relevant for our present focus is that it enables the formulation of a specific *animal* sensorimotor organization that diverges from standard notions of input and output (Keijzer 2015).

The ASMO is a sensorimotor organization that is sensitive to the patterns of changes in stress across its contractile surface—referred to as the animal’s Pantin surface—in a way that reflects both self-induced and externally induced disturbances of these patterns. This organization, it is proposed, provides a way for macroscopic intercellular events (patterns across the Pantin surface) and (intra)cellular events to ‘talk’ to one another. Rather than being limited to localized reactions to environmental stimulation, this organization unifies a multicellular organization allowing it to deal with patterns at this bodily level. Rather than being limited to *re*-actions, this organization is intrinsically active and initiates its own changes of shape creating its own feedback of induced (reafferent) changes as well as exafferent changes that convey information about external features. This ASMO nicely fits the view of the sensorimotor organizations as influenced by work in ecological psychology (Chemero 2009; Turvey and Fonseca 2014) and sensorimotor theories (Hurley 2001; O’Regan and Noë 2001).

To develop the ASMO notion in more detail we combine it with current knowledge about epithelia and relate it to the basic animal configurations of Porifera and Cnidaria. For this discussion, we want to stress the relevant theoretical posits that will be discussed.

First, the ASMO relates to a particular organization, a way of putting a system together, that is not strictly tied to any specific historical scenario. It is a proposition about how individual animals might have functioned and potentially still function. For this purpose it builds on theorizing within embodied cognition that stresses the essential role for self-generated movement and the perceptual feedback this generates (e.g. Hurley 2001; O’Regan and Noë 2001; Barrett 2011). The evolutionary importance of these organizational features is plausibly cast as providing various organizations on which selection might have taken place (Calcott 2009).

Second, the ASMO is consistent with Input–Output (IO) views, such as the ECT. It focuses on different issues by highlighting the (internal) organismal organization on which much IO behavior depends (Jékely et al. 2015a). As said above, there can be some tension between the two however. For IO views like the ECT, accessing environmental information through senses is basic. The IC inspired ASMO notion suggests that a highly complex and specific multicellular organization provides a precondition for the ECT when this is applied to the macroscopic environments of modern animals. We will return to this possible implication in the final section.

Third, while the ASMO is formulated in the context of the evolution of early nervous systems, we do not focus here on the actual involvement of a full nervous system. What counts most is a spatially extended effector surface, electrical (or maybe chemical) signaling across that surface, and the various ways in which the patterns of tension differences across that surface might feed back to and influence the processes that initiate and are responsible for that patterning signaling. Nervous systems are thus not a precondition for the ASMO in this account, but rather a consequence of its presence and use.

To organize the discussion of the ASMO notion in this paper, we use five theoretically grounded conditions concerning the kind of living organization that can be expected to yield a basic ASMO. Thus we take the ASMO notion to subsume:

1. a multicellular body, constituting an ‘inner space’ or domain, which is differentiated from the body’s ‘outer space’ or environment.
2. the presence of contractile epithelia.
3. complex, standardized body architectures.
4. sensitivity to tension and stress at the level of (intra) cellular processes.
5. reversible, contraction-based changes in body-shape.

The first four are central features of the ASMO notion as formulated originally by Keijzer (2015). The fifth is an addition that addresses the issue how contraction and ciliary motility can be related in a basic ASMO. While the ASMO eventually turned into the foundation of muscle-based motility, such motility itself derives from reversible changes in body-shape, whether used for motility or not. Below, we discuss how these posits line up nicely with various central features exhibited by epithelia, and allow us to extend the relevance of features that are already closely studied in developmental biology to the ASMO.

## Epithelia and their features

Epithelia are the key to the animal multicellular organization.^1^ Tyler (2003) argues that epithelial cells are the default eumetazoan cell state, while epithelia provide the central ingredient for developmental signaling as well as the actual construction material and developmental scaffolding for much of the animal body (Tyler 2003; Holland 2011). Some authors compare embryogenesis to origami folding: the flexible two-dimensional epithelial sheets bend and fold themselves in specific ways and in elaborate sequences, eventually producing the amazing three-dimensional structures that constitute the animal body (Holland 2011; Mao and Baum 2015). Epithelia, together with the cells that constitute them, are thus central to the animal organization and make it different in significant ways from non-metazoan multicellular organizations (Tyler 2003; Arnellos and Moreno 2016). In this section, we give a quick overview of some central features of epithelia with a general eye to their relevance for the evolution of the ASMO.

An epithelium is a two-dimensional sheet-like tissue that consists of polarized cells anchored in an underlying extra-cellular basal membrane (or lamina), which is part of the underlying extracellular matrix (ECM). The ECM is a continuum of collagen fibers and fibrils that integrates and reinforces the epithelium making it more resistant to tension and provides a stable setting for the epithelial cells (Timpl 1996). Epithelial cells have an outward-facing apical side with a primary cilium, a basal side connected to the basal membrane, while they are also connected to adjacent epithelial cells through a variety of junctions (Tyler 2003; Ingber 2006; Magie and Martindale 2008).

Epithelial cells have various types of junctions, which can differ between the various lineages. We discuss the ones that are most general and functionally relevant for the ASMO. *Adherens junctions*—mainly constituted by proteins of the cadherin family—provide strong physical connections between cells, tethering them together, while also making contact with the intracellular cytoskeleton, which is composed of actomyosin networks, microtubules and intermediate filaments (Magie and Martindale 2008; Engler et al. 2009). In addition to adherens junctions there are also integrin-based adhesion complexes—*focal adhesions*—positioned at the basal side of the cell, connecting it with the basal membrane. Focal adhesions are highly sensitive to forces imposed on the ECM (Engler et al. 2009; Geiger et al. 2009). A second central junction type consists of *occluding junctions*. These seal off the space between the epithelial cells for diffusing molecules (Skaer and Maddrell 1987; Magie and Martindale 2008), which creates molecular gradients that enable intercellular signaling and electrical differences (Cereijido et al. 2004). Occluding junctions allow epithelia to close of extracellular spaces from the general environment turning them into compartments or containers with a distinct molecular composition. Such compartments provide stabilized environments where various physiological processes can proceed without being disrupted by environmental changes.

Another central characteristic of epithelial cells is their capacity to move, to change shape and to respond to and actively impose mechanical stress. Epithelial cell use their actomyosin cytoskeletons to produce and resist such stress, which can travel through their adherens junctions and focal adhesions to both neighboring cells and the ECM (Wozniak and Chen 2009). This self-induced and transferred motility also drives the folding patterns during development. The mechanical forces generated by the cells’ cytoskeletons are essential for developmental processes such as proliferation, differentiation and spatial rearrangements (Ingber 2006; Chen 2008). Mechanical forces are now recognized as being involved in processes like wound healing (Röper 2013) and differentiation of stem cells (Nava et al. 2012), but they also influence gap junction coupling (Salameh and Dhein 2013) and the activity of ion channels (Sachs 2010). Thus mechanical forces are a key feature that influences epithelia at scales that range from the tissue level to the biochemical signaling within and between cells.

Epithelia further provide the context for intercellular signaling that together make epithelia the self-folding origami virtuosos capable of building the complex animal bodies we witness today. Epithelia provide a reliable scaffold to guide diffusing morphogens that initiate developmental processes like cell differentiation and various morphogenetic movements at specific places in ways that fit the global patterning process. Sensitivity to the spatial lay-out enables e.g. axial differentiation, cell-type specification and regional tissue patterning (e.g., Davidson and Erwin 2006; Newman 2012; Newman and Müller 2010). The result consists of developmental programs based on both intra- and intercellular signaling mechanisms that mobilize and drive morphogenesis.

In addition to genetic signals and soluble morphogens, such developmental processes are also regulated by the mechanical forces mentioned above that act as “mechanochemical actuators” (Kim et al. 2014). Given the relevance for the ASMO notion it is worthwhile to highlight this component of developmental patterning:

mechanical forces play a central role in morphogenesis and tissue patterning, and […] physical cues are as important as chemical factors for developmental control throughout virtually all stages of embryogenesis. Although the contribution of physical forces to cell and tissue deformation in the embryo has been recognized for over a century, it is only recently that mechanical stresses have been shown to function as informative signals that produce specific changes in molecular biochemistry and gene expression through the process of cellular mechanotransduction (Mammoto and Ingber 2010, p. 1416).

In this context, Geiger et al. (2009) talk about “environmental sensing” by epithelial cells as they are sensitive to and integrating a wide array of chemical as well as physical cues that act on the focal adhesions. Connections through adherens junctions also ensure that stress induced in one cell spreads to adjacent cells, which provides a way to integrate activity at an intercellular scale, as also occurs by supracellular actomyosin cables (Röper 2013). Such sensitivity is not only passive as cells themselves also respond actively to applied forces, which can lead to “an autoregulatory feedback control that ensures the robust sculpting of cells and tissues during developmental growth and morphogenesis” (Mao and Baum 2015, p. 98).

Taking the relevance of mechanotransduction into account, Ingber (2006) states that prestress at all levels of the tissue organization ensures that various molecular scale mechanochemical transduction mechanisms proceed simultaneously and produce a concerted response. He argues that this should be understood in terms of a tensional integrity (tensegrity) architecture that consists of opposing tension and compression elements that balance one another by providing an internal resting tension that stabilizes the whole structure (ibid., p. 821). In this way, a global and mutual coordination of the many separate intra- and intercellular events at a wide variety of size scales could be achieved.

Finally, we should not forget the ongoing relevance of electrical signaling, where epithelial conduction is an important predecessor or parallel organization for nervous systems (Mackie 1970, 2004a; Meech 2015). We should also mention more recent developments about the developmental and regenerative relevance of bioelectrical signals (e.g. Tseng and Levin 2013; Pai et al. 2015). According to Levin (2013), the spatiotemporal gradients of endogenous transmembrane voltage potentials serve as instructive patterning cues for large-scale anatomy, providing organ identity, positional information, and prepattern template cues for morphogenesis. The promise here is that morphogenesis actually can be manipulated by these patterning signals.

To conclude, epithelia are an extremely versatile tissue configuration that has been intensely studied for its role in morphogenesis and development. In the following, we will apply these findings and theories about epithelia to further develop and clarify the notion of the animal sensorimotor organization.

## Epithelia and the animal sensorimotor organization

The ASMO in its most basic form has been cast as an epithelial organization. The initial formulation took place in the context of early nervous systems and most attention was given to studies on electrical signaling by excitable and contractile epithelia (Josephson 1985; Mackie 1970, 2004a; Meech 2015). However, epithelia are more versatile than this, while the ASMO notion itself stands for a sensorimotor entity rather than a signaling or information processing device. For this purpose, we will discuss the ASMO conditions listed earlier to see how they connect to this wider array of epithelial features.

### The ASMO notion subsumes a multicellular body, constituting an ‘inner space’ or domain, which is differentiated from the body’s ‘outer space’ or environment

Having a multicellular body is an obvious but also wide precondition for the ASMO. Without it there would be no multicellular organism at al. As discussed above, epithelia provide the key ingredients for building an integrated body. The flexible and easily to manipulate ‘origami’ sheets provide a good construction material, the two- and later three-dimensional structure provides a scaffold along which chemical signals can travel and where differences in membrane potentials can have their specific signaling effects for development.

This precondition becomes less obvious when attention is given to the word *body* and the claim that multicellular plants and fungi do not have bodies like animals have. The word ‘body’ conveys the presence of a specific integrated unit, for which the ASMO notion is set to provide an organizational story. This organizational story can also be linked to an ecological context where such bodies make evolutionary sense.

The ASMO is cast as a particular organization that enables sensorimotor relations at a multicellular level that are sensitive to macroscopic, changeable environmental stimulus arrays, such as in touch and vision. The question why, from an evolutionary perspective, it would be relevant to have such an ASMO is not addressed. It is similar to work on disentangling possible eye configurations in some lineage where the evolutionary relevance of eyes can be assumed. Still, the concept of a body has evolutionary features that connect to the ASMO in a plausible way.

The most relevant feature here is probably *macrophagy*, which means feeding on large food items. The feeding systems of animals take three main forms (Sperling and Vinther 2010). Sponges have a water canal system and water is pumped through the body where bacteria are filtered out and digested intracellularly, making this a form of microphagy. The only described species of the placazoa has a ventral mucociliary sole (Arendt et al. 2015), which consists of an extracellulary space between the underside of the organism and the surface it is positioned on. Barring some weird exceptions, other animals have an internal gut in some form or other, where large food items are digested inside the body but outside of the cells. Sperling and Vinther call the gut possibly the greatest metazoan innovation, which has been the key to the Cambrian prey-predator interactions and even the Cambrian Explosion (see also Arendt et al. 2015).

Macrophagy provides an ecological context where an ASMO becomes highly relevant. Foremost, for a mucociliary sole or a gut to work, the body must be positioned in relation to food items, which involves a sensorimotor process that actively and precisely manipulates the environment at the bodily scale. Interestingly, this life-style also involves the active manipulation of the body itself including regulating the treatment of ingested food, excreting waste, and maintaining a suitable internal homeostasis at a multicellular level by means of complex physiological processes. This fits in with the focus on internal coordination that is behind the ASMO notion and where the classic differentiation between inner physiology and external behavior becomes more diffuse as both constitute their own environments (Jékely et al. 2015a). In contrast, macrophagy need not involve any complex—heterogeneous—environment as long as the food items are plentiful and easy to find for a motile organism.

Epithelial characteristics are also relevant to generating the features relevant for macrophagy, including sealing off internal compartments for various functions (Rosslenbroich 2014). When the contents of such compartments can be buffered against a changing environment the concept of homeostasis can be extended to the multicellular organism level and in this way epithelia play an essential physiological role both during and after development (Skaer and Maddrell 1987; Leys et al. 2009).

### The ASMO notion subsumes the presence of contractile epithelia

Many cnidarians have a contractile apparatus that consist to a large extent of contractile epithelia. As such, it is an unproblematic assumption to take such epithelia as a starting point for the ASMO. What the broader view on epithelia adds however is that contractility is a much more general and fundamental aspect of epithelia. Considering the essential role played by cellular contractions and tissue deformations as a part of development, it becomes clear that contractility constitutes a key feature of the animal organization from the very start. In addition, manipulating patterns of differentiated contractions across epithelial surfaces is a basic feature of many developmental processes and thus provides a long-standing counterpoint to manipulating much more fleeting and short-lived patterns of contraction across a contractile surface for reversible movements.

### The ASMO notion subsumes complex, standardized body architectures

The ASMO notion is centered on a standardized Pantin surface: the total contractile surface that is available in a particular species (and ultimately an individual organism) for initiating motility (see also Arnellos and Moreno 2015, 2016 for the role of motility in multicellular agency and organismality, respectively). The idea is that the fast reversible movements made by animals can only be organized in a way that is reliable and robust, when they play out across a surface that remains stable in size, topology and extension across many life-time occurrences of these fast movements. Fast and reliable motion will be severely hampered if your body keeps changing its topology. A stable body topology can change shape, sometimes in spectacular ways such as by squid, but the cellular tissue arrangement remains the same during those movements. In addition, a reliable controlling structure in itself involves some form of standardization. Thus, having a stable contractile platform and controlling structure—a nervous system—enables the initiation and maintenance of stable forms of patterning across this surface, which, in turn, leads to increasingly complex and useful forms of outward behavior.

While this is a speculative claim, it is corroborated by the fact that animals that rely on muscle-based motility show standardized body topologies built around collections of muscle groups. It is noteworthy that both Porifera and Placozoa, phyla without clear muscle tissue or a nervous system often lack a standardized body topology. Sponges can take many forms, often adapting to local circumstances, while *Trichoplax adhaerens*, the only named placozoan species, has no clear symmetry, nor a differentiated anterior-posterior or left–right body-axis (Eitel and Schierwater 2010). This precondition of a standardized body architecture implies that the ASMO requires complex forms of development, which is precisely one of the mainstays of an epithelial organization (Arnellos and Moreno 2016).

### The ASMO notion subsumes sensitivity to tension and stress at the level of (intra)cellular processes

Again this is a feature that has now been shown to be essential to developmental patterning. In addition to genetic programs and morphogens, mechanical forces also play a central role in development: The developmental differentiation of a multicellular organization is sensitive to mechanical stresses. Thus, positing a faster and reversible form of tension generation and tension sensitive coordination is more a matter of tweaking and adding to an organization that is already present than adding something totally new. A global and mutual coordination of many compression and tension components is a plausible starting point. In this context, it is interesting to note how some authors use the phrase of *sensing* to describe the way in which cells within a bodily environment interact with this environment (e.g. Geiger et al. 2009).

### The ASMO notion subsumes reversible, contraction-based changes in body-shape

Animals have two major options for becoming motile. They can use some form of muscle contraction or they can use beating cilia. Cilia are omnipresent in small organisms, including many animals. Contractile tissue is also used by small animals, sometimes in conjunction with cilia, but it is essential to enable motility for large animals, large meaning here more than a few millimeters (barring some special cases). The ASMO notion stresses the contractility and feedback provided by a Pantin surface, but without a clear role for cilia-based motility. Still, the neural control of ciliary motility must also have been an important factor for early nervous systems (e.g. Jékely 2011; Jékely et al. 2015b). The discussed work on epithelia provides some options to bring these ideas closer together.

From the presented findings, it becomes clear that contraction is a basic feature of epithelia that derives from the intracellular actomyosin cytoskeleton as well as intercellular adherens junctions. Contraction is essential to development, but it is also used in animals at a behavioral level without directly involving motility. Sponges use (slow) body contractions as a defense mechanism (see below), while placozoa change body shape by contraction (Pearse and Voigt 2007). Of the animals with recognizable muscle, ctenophores (comb jellies) use them to maintain body shape while forward movement is achieved by cilia, and many different worm-like animals also move forward by cilia while steering by muscles that orient the front end of the body (Tyler and Rieger 1999). Taken together, these findings suggest an option where an ASMO first arose as part of an organization that modulated body shape rather than as a ‘behavior machine’ to use Pantin’s phrase. As long as the operation of patterning a Pantin surface together with bodily and environmental feedback is present, these cases still constitute an ASMO.

At present these options remain speculative, but they suggest how there could have been a gradual approach from basic and general epithelial features to the origins of the typical contraction-based animal organization that is here cast as the key factor behind the animal sensitivity to the many fleeting aspects of their macroscopic environment.

To conclude more generally, for all five conditions discussed here there is a good fit between the requirements of the ASMO and the standardly available features of epithelia and the developmental and regulative processes in which they are involved. This suggests that internal organization—in the form of an epithelial organization—and bodily complexity can be cast as the initial key-factor for the origin of the ASMO (see also Arnellos and Moreno 2016). In this case, environmental complexity may have played a relatively minor role. Before turning to possible ways to resist this conclusion by the ECT, we will first discuss Porifera and Cnidaria, returning to this issue in the final section.

## Two model animal organizations: Porifera and Cnidaria

Here we position the ASMO notion with respect to the characteristic features of two basic animal phyla: Porifera and Cnidaria. These phyla arguably straddle the divide between animals with and without both a standardized body and an ASMO. In this way, we aim to show how different forms of an epithelia-based multicellular organization can exist and how the ASMO notion can be positioned in this landscape.

As a word of warning, the discussion in this paper targets a relatively abstract level of description. It aims to explain how a collection of individual cells and their products can constitute an organization that is sensitive to and able to deal with increasingly fleeting and changeable macroscopic aspects of the multicellular body and the environment. It cannot be taken for granted that anything living today resembles the various intermediate forms that must have lived so many years ago. Still, Porifera and Cnidaria are representatives of basic animal forms and a lot can be learned from them. The question asked here is whether they do or do not exemplify the ASMO notion and what we can learn from the answer.

### Porifera

Porifera (sponges) are sessile aquatic animals that are found in almost all benthic habitats. Sponges have no clear body symmetry and can take many different shapes. As mentioned above, sponges have no gut but an extensively branched water canal system that extends throughout their bodies. Water is taken into the sponge body through tiny holes (ostia) in pore cells in the surface epithelium from where it goes to chambers with choanocytes that, operating like cilia, generate this feeding and respiratory current. Finally the water is expelled through a large opening, the osculum. Sponges feed on picoplankton, such as bacteria and viruses, by filtering the water in a highly efficient way. Food uptake takes place within cells, which is a form of microphagy.

Despite a long-standing discussion, sponges can be considered as epithelial organisms (Leys et al. 2009; Leys and Riesgo 2012). Sponge epithelia function as both sensory and contractile tissue, and they are the primary conduits of behavior coordination (Nickel et al. 2011; Leys and Hill 2012; Leys 2015). Sponges lack nerve cells and so far no electrical signaling has been witnessed within cellular sponges (Leys 2015).^2^ The propagation of signals along the whole sponge can take from 30 min to a full hour, although individual pores can be closed in some tens of seconds. In her review, Leys (2015) names juxtacrine signaling by chemical signals from one cell to the next along the epithelia as the most likely candidate.

Given the notion of an ASMO, the contractile behavior of sponges and its coordination are most relevant. Sponges use contractions in response to touch and to regulate the flow of water through their bodies and expel obstructions (Elliott and Leys 2007). They have contractile cells called actinocytes that come close to some form of smooth muscle (Elliott and Leys 2007). How these contractions are coordinated is not fully clear, but this seems to depend on this juxtacrine chemical signaling (Leys 2015). In any case, very slow contraction waves can be observed that are in many ways similar to peristaltic contractions (Elliott and Leys 2007). Sponges tune the canal diameter to a high degree to modify water flow (Leys 2015). Sponges can expel sediments by contracting and expanding the osculum and canals and pumping out water at a velocity that carries waste products some distance away (Leys 2015). Uptake of unwanted particles can be limited by the contraction of the ostia and the canals, which can be triggered for example by turbulence or the amount of sediment. All in all, it can be concluded that “sponges have, without nerves or true muscle, evolved a way of coordinating contractions of cells to generate an effective mechanism of controlling water flow” (Elliott and Leys 2007, p. 3746).

Given this general description, to what extent do sponges exhibit an ASMO? Sponges fulfil some but not all of the conditions discussed above. The easiest cases are the presence of contractile epithelia and body contractions, which are both a clear yes. The condition concerning sensitivity to tension at the (intra)cellular level is also plausibly fulfilled, considering the sensitivity to canal width and the developmental processes involved in building the sponge body. The other two are a different matter however. Sponge bodies are not standardized in the way eumetazoan bodies are. The bodies of individuals often adapt during development to the specific local circumstances and are in this sense more like plants that can also take many individual shapes even when their local characteristics are typical for the species involved. The contractile surface is subsequently also not standardized and not suitable for quick reversible changes of shape. Finally, there is a less than clear distinction between an inner and outer body space. The water canal system is pervasive and involves a very high throughput of material that flows right through the body making it part of the environment rather than the body itself. In contrast, the mesohyl, the sponge body’s inner space that is actually closed off from the environment and molecularly controlled, seems to function mainly for support.

All in all sponges do not have an ASMO as formulated above. But, maybe surprisingly, it looks like a near miss: Sponges do have a contractile body and various (though slow) feedback processes to control these contractions. When compared to clear examples of an ASMO, such as the Cnidaria discussed below, these differences look not exceptionally large and can be summarized as a difference in speed, integration and specificity. To become sensitive to often fleeting environmental happenings an ASMO must operate much faster than what is seen in sponges. Speed is also needed to integrate any behavioral response for the whole animal and making it specifically tailored for different behavioral tasks. To clarify these claims, we will have a quick glance at Cnidaria.

### Cnidaria

Jellyfish are the best known Cnidaria and we will use them here as their representatives. Jellyfish are free-swimming marine animals consisting of a gelatinous bell and trailing tentacles. Their body architecture is characterized by a central digestive compartment, two epithelial layers and mesoglea in between. They have muscle, a nervous system—as well as conductive epithelia—and simple sensory organs distributed radially around the body. Jellyfish move forward by a form of jet propulsion brought about by contracting their bell-like bodies and expelling the water from the central cavity, the elastic mesoglea extends the bell again (see Mackie 2004b; Satterlie 2002). Cnidarians regulate their physiology by means of neuroendocrine cells that synthesize peptide-signaling molecules (acting as neurotransmitters or neurohormones), which are dispersed primarily through diffusion. Almost all neurotransmitters, neurohormones and non-neuronal hormones are present in Cnidaria and they are central in regulating developmental and physiological processes, such as the induction or inhibition of neural differentiation, neurogenesis, and oocyte maturation, morphogenesis and spawning (e.g. Koizumi 2002; Takahashi and Hatta 2011). In general, jellyfish have the capacity for diverse contraction-based motile interactions with the environment that are mainly manifested as swimming with increased maneuverability combined with directional movements of various parts of their body.

Jellyfish and cnidarians more generally exemplify a full ASMO: a multi-cellular body with an inner space, contractile epithelia and even muscle, a complex standardized body architecture, sensitivity to tension can be assumed, and changes in body shape as well as motility are achieved by body contraction. Jellyfish thus show that a clear and recognizable ASMO is present at a very ‘basic’ level, and can be achieved with limited neural coordination. This basic level involves a clearly standardized and differentiated body, integrated in a way that enables fast reversible movements that are well coordinated in feeding, escaping and other behavior. Organizationally, the neural and other bodily complexities that evolved within the bilaterians can be interpreted as filling in the new option space that became available when the ASMO first arose.

Another interesting issue derives from a comparison with sponges as discussed below. When it comes to the notion of an ASMO, sponges look like they already have most of the ingredients, while just adding ‘speed, integration and specificity’ would suffice to make this transition. An interesting question here is whether the sponge feeding strategy provided an impediment to making the step towards an ASMO. Macrophagy as a lifestyle could be the central factor behind the evolution of an ASMO. Even when feeding on non-moving food particles, ingesting them involve comparatively quick and precise manipulations, which requires a specialized body, which in turn requires a specialized developmental processes (Arnellos and Moreno 2016). In this case, one can envision a clear evolutionary route that leads from non-predatory macrophagy to a basic ASMO and a clear role for coordinative processes as a key feature behind this evolutionary process.

## Bodily complexity meets environmental complexity

The notion of a specific animal sensorimotor organization or ASMO provides a central preadaptation for the subsequent evolution of complex active animal bodies that kick started the Cambrian Explosion of smart and fast animal life on this planet. Coordinating contraction-based effectors provided a way for macroscopic bodily and environmental features to systematically influence microscopic (intra)cellular processes, integrating them in a way that did not happen in other multicellular organizations. Once such an ASMO is present—such as in the case of cnidarians—and when there is space for the further developmental explorations of complex body forms—where the bilaterians must have had better cards—the Cambrian Explosion starts to look like a necessary consequence.

While the last remark is pushing too far, we do think that the ASMO notion is an important conceptual tool to think about the processes that gave rise to the first complex adaptive bodies, as well as about the ways in which brains, bodies and environments work together even now. We think that the ASMO concept adds an important organizational view to the current discussion on the origins of early nervous systems (see e.g. Arendt et al. 2015; Arendt et al. 2016; Jékely et al. 2015b; Moroz 2015), and also to current debates in cognitive science on embodied intelligence (see e.g. Chemero 2009; Barrett 2011; Turvey and Fonseca 2014).

In addition, the ASMO notion relates to the ECT, reflecting an internal coordination view, while the ECT stresses adaptation to environmental complexity. We have argued that epithelia—the fundamental building block of the animal organization—provide a congenial starting point for deriving the ASMO. The kind of evolutionary problems encountered in this reading relate to the internal coordination of development, behavior and physiology. The environment in this reading is cast as comparatively homogeneous (simple in ECT terms), not requiring complex cognitive features, except an integrated body that is capable of providing ways to digest large food particles lying around. The relevance of environmental complexity is comparatively small in this scenario.

For the ECT, there are various ways to resist this critique. Obviously, the account of the ASMO as presented here is preliminary and whether it will be accepted depends on further theoretical and empirical work. Any strong implication for the ECT will depend on how the ASMO notion fares. Another possibility we see is to stress macrophagy as a central environmental challenge that drives the evolutionary events discussed above. The problem here has been discussed and consists of the lack of environmental complexity involved in just eating larger food items. ECT might be defended here but this will require additional work to show how and why macrophagy entails environmental complexity.

## Footnotes

1 There is discussion whether all metazoans (animals) have genuine epithelia or only eumetazoans, which would exclude the sponges (Tyler 2003; Magie and Martindale 2008; Leys and Riesgo 2012). Here we apply the concept of epithelia to all animals.

2 Glass sponges provide an exception that we will not discuss here. Their way of electrical signaling looks like a derived feature.
